# How to improve emotional regulation in breast cancer survivors? A psychological intervention

**DOI:** 10.3389/fpsyg.2024.1443635

**Published:** 2024-09-13

**Authors:** Valeria Sebri, Giulia Rosa Policardo, Gabriella Pravettoni

**Affiliations:** ^1^Applied Research Division for Cognitive and Psychological Science, IEO, European Institute of Oncology IRCCS, Milan, Italy; ^2^FORLILPSI Department (Education, Languages, Intercultures, Literatures and Psychology), University of Florence, Firenze, Italy; ^3^Department of Oncology and Hemato-Oncology, University of Milan, Milan, Italy

**Keywords:** breast cancer survivors, emotional regulation, psychological intervention, positive body image, quality of life

## Abstract

**Objective:**

Psychological interventions are pivotal in enhancing the Quality of Life for breast cancer survivors, with a primary focus on addressing affective and cognitive challenges through group discussions among those diagnosed with the disease. While the influence of Body Image on overall well-being is well-documented, research on interventions specifically designed to address Body Image concerns in this demographic remains scarce. The present study aimed to fill this gap by evaluating the outcomes of a psychological intervention focused on fostering a positive Body Image among 25 breast cancer survivors.

**Method:**

Participants were divided into an experimental group, which received the intervention (*n* = 13), and a control group that did not receive any psychological support (*n* = 12).

**Results:**

Our findings highlight significant disparities in emotional regulation strategies, specifically cognitive reappraisal and expressive suppression, with the intervention group reporting enhanced emotional regulation. Contrary to initial hypotheses, the analysis unveiled statistically significant differences in both negative (social physique anxiety) and positive (functionality appreciation) body image dimensions, indicating elevated levels of social physique anxiety and reduced functionality appreciation among intervention participants.

**Conclusion:**

The total results may suggest that the intervention, while effective in enhancing emotional regulation, heightened awareness of body image issues, leading to increased social physique anxiety and diminished functionality appreciation. The paper further discusses practical implications arising from these insights.

## 1 Introduction

Breast cancer survivors have to deal with cognitive, emotional, and relational issues over years (Sebri et al., 2022). At the time of diagnosis and oncological treatments (e.g., surgery, chemotherapy, radiotherapy, and hormonal therapy), the body can be seriously damaged due to undesirable side-effects. For example, the loss of one or both breasts, scarring after surgery, and hair loss are some of the possible bodily signs after oncological treatments (Fioretti et al., 2017). Similarly, surgical interventions, such as mastectomy (with or without breast reconstruction), impact on the body and women’ perception about it. Literature evidenced that radical breast removal can significantly impair satisfaction toward the body; in this regard, radical procedures lead to permanent marks on the body, which increase less body satisfaction (Zhou et al., 2020). Accordingly, an incurring premature menopause and/or infertility strongly impair the perception of femininity.

Consequently, Body Image (BI) strongly changes during and after breast cancer in both the short and long-term (Jabłoński et al., 2019). As a definition, BI is “internal representation of one’s own outer appearance” (Thompson et al., 1999, p. 4). Considering the bodily representation’ field, BI is not only the evaluation of the physical appearance (in terms of shape and dimension), but it refers to the mental representation of the body and its related emotions (Lewis-Smith et al., 2018; Sebri et al., 2021b). The perception of a negative BI is closely linked to the escalation of negative emotions, including anxiety, depression, shame, and distress. These emotional states not only detract from an individual’s Quality of Life, but also have a tangible impact on the adherence to treatment protocols and, consequently, on health outcomes. This way, addressing emotions is fundamental to deal with breast cancer issues. Additionally, BI encompasses attitudes toward the body involving two main factors: investment in appearance and self-evaluation. A positive BI can be indeed the results of a good perception of the own aesthetical appearance. At the same time, perceiving disparities between BI and cultural ideals regarding physical appearance impact on emotions strongly (Cash et al., 2004; Cash and Smolak, 2011). A negative perception of BI can enhance the tendency to self-scrutiny their own bodies, highlighting a self-objectification process (Fredrickson and Roberts, 1997). Therefore, perceiving differences from cultural stereotypes increases shame and the fear of others’ judgments, enhancing the desire to stay alone or with significant others only (Sherman et al., 2018). However, intimate and sexual relationships could be impaired too due to the perception of sexual unattractiveness (Male et al., 2016). This way, BI is closely intertwined with identity, affecting self-esteem and social relationships (Falk Dahl et al., 2010). Over the year, numerous psychological interventions addressed BI, as showed in a systematic-review and meta-analysis by Sebri et al. (2021a). This study stated that psychological interventions are effective in enhancing BI among breast cancer patients and survivors. Findings suggest that these improvements are not tied to a specific intervention modality but multiple variables likely contribute to the obtained benefits across the studies reviewed. Additionally, the interventions were conducted by psychologists who addressed the various psychological, emotional, and social aspects of body image. Accordingly, one of the effective studies implement for the BI promotion was conducted by Lewis-Smith et al. (2018), proposing the “Set Your Body Free” intervention. The present program was based on an aetiological approach focused on risk factors for body dissatisfaction among women. Therefore, the psychological intervention targeted factors related to sociocultural appearance pressures as well the internalization of aesthetical ideals, which have been addressed also in our study.

Recognizing this, contemporary research has increasingly focused on the relevance of emotions in breast cancer patients and survivors and, as a consequence, on the development and implementation of cognitive emotional regulation strategies. These strategies aim to address and mitigate the dysfunctional emotions that can significantly hinder treatment adherence and negatively influence recovery and well-being (Kovač et al., 2020; Supriati et al., 2021). Evidence suggests that breast cancer survivors exhibit a heightened sensitivity to interoceptive sensations, potentially amplifying fears related to the cancer’s return (Harris et al., 2017). Such an increased focus on internal sensations, previously overlooked, becomes a significant source of anxiety. Consequently, this heightened state of alert may lead survivors to a diminished capacity to interpret internal cues accurately, reducing their interoceptive abilities. This pervasive and unbalanced psychological state contributes to an increase in emotional dysregulation, a critical concern among breast cancer survivors. Emotion regulation is defined as the process of individuals managing, monitoring, and adjusting their emotional experiences and expressions (Gross and John, 2003). Two of the main key factors of emotional regulations are expressive suppression and cognitive reappraisal strategies. Firstly, expressive suppression refers to the process of inhibiting the outward signs of one’s internal emotional states. It is a response-focused strategy, employed after an emotional response has been generated, with the aim of suppressing the behavioral expression of emotion (Gross and John, 2003). Therefore, it is a significant psychological factor associated with depressive symptoms among patients with breast cancer (Li et al., 2015). Cognitive reappraisal involves efforts to reframe and gain a new perspective on an emotional situation to alter its previous and dysfunctional emotional significance. Reappraisal acknowledges that our emotional reactions stem from our thoughts, thus it is needed to reframe our appraisal to enhance well-being (McRae, 2016). On the contrary, emotional dysregulation refers to the diminished ability to manage and respond to emotional experiences in a healthy manner. This issue is pivotal, as it underpins a range of psychological challenges that can impede recovery and adversely affect survivors’ Quality of Life (Fritzson et al., 2024). Those who possess greater proficiency in handling and expressing their emotions typically exhibit better adaptation to the external context, leading to increased life satisfaction and higher self-esteem (Gross and John, 2003; Stanton et al., 2000). Furthermore, studies suggest that effective emotion regulation is associated with perceived post-traumatic growth (Karimzadeh et al., 2021). To sum up, a positive BI can depend on women’s experience of breast cancer and the possibility to address its related issues after cancer, in terms of emotions and dysfunctional behaviors. It is paramount to highlight the relevant role of the body after cancer and its influence on breast cancer survivors’ emotions and relationships. Over the years, several psychological interventions deal with breast cancer issues, with a specific focus on BI (Sebri and Pravettoni, 2023). Most of the present programs were based on interpersonal, cognitive-behavioral, psychosocial, and emotional expressive interventions. In particular, body compassion intervention can promote a new and kind approach to one’s body, which represents a protective factor to enhance positive embodiment and protection against body shame (Burychka et al., 2021).

In the present study, we hypothesize that, following the intervention, the experimental group will demonstrate a better body image evaluation and improved emotional regulation compared to the control group. Specifically, participants in the experimental group are expected to exhibit higher level of positive body shifts in their perceptions of BI, more effective emotional regulation skills in contrast to those in the control group. In particular, we will expect that: (Hp1) The experimental group will demonstrate increased positive aspects of body evaluation, such as functionality appreciation, and body compassion, as well as decreased negative aspects related to physical appearance, such as social physique anxiety and body dissatisfaction, compared to the control group; (Hp2) The experimental group will show improved emotional regulation (in terms of lower expressive suppression and higher cognitive reappraisal) compared to the control group.

## 2 Materials and methods

### 2.1 Procedure

The study was conducted in accordance with the Declaration of Helsinki and was approved by an Ethics committee by the University of Milan (n. 45/23). Breast cancer survivors received an invitation to participate voluntarily in the present psychological intervention through a mailing list. At the same time, women were recruited via social networks (e.g., Facebook), where breast cancer survivors found an announcement to participate in this project. Thus, a self-selected method has been applied first. Then, researchers select participants in line with the following inclusion criteria: (1) women who are 18 years old and older; (2) having received a breast cancer diagnosis and having completed oncological treatments at least 5 years ago. This is in line with the definition by the National Coalition for Cancer Survivorship, which establishes that a cancer survivor is a patient who has been disease-free for 5 years (De Fusco and Chlebowski, 2009; Marzorati et al., 2017); (3) the lack of current oncological treatments; (4) understanding and speaking of Italian language in an appropriate way. Women exhibiting psychological impairments, an inability to comprehend the study’s contents and objectives or to independently provide informed consent, were excluded from the participant pool. Breast cancer survivors who met inclusion criteria receive an email with an information sheet and an informed consent to be signed. Moreover, researchers send a ZOOM link to participate in the online psychological sessions. After the four psychological sessions, a Qualtrics link with a battery of questionnaires was sent to the women who had participated in the intervention in its entirety. Specifically, participants were asked to fill in socio-demographic data (e.g., age and the year of cancer diagnosis) and self-administered and standardized questionnaires.

Women who participated in the psychological sessions (experimental group) and those who were not involved in the psychological sessions (control group) filled in the questionnaires. Any personal information was deleted to guarantee participants’ anonymity. Following a self-selected method of recruitment, experimental and control groups have been formed as follows.

#### 2.1.1 Experimental sample

After receiving the email or reading the online post, 15 breast cancer survivors voluntarily responded to the study invitation. However, only 13 women were fully engaged in the psychological intervention program and completed all the questionnaires. They had an average age of 48 years, with a standard deviation of 7.65, highlighting a middle-aged demographic. Geographically, the participants predominantly hailed from Northern Italy, constituting 69.2% of the sample, while 23.1 and 7.7% resided in Southern and Central Italy, respectively. The educational attainment within the group was diverse: 25% possessed a high school diploma, 12.5% had completed undergraduate studies, 25% had obtained a master’s degree, 12.5% held a doctoral degree, and the remaining 25% had completed a master’s or other specialized postgraduate training. In terms of marital status, the sample was varied, with 30.8% single, 53.8% married, 7.7% separated, and 7.7% in a cohabitation arrangement. Employment status was high across the group, with only one individual reported as retired. Questions about family dynamics revealed that 69.2% of the participants were parents to one or two children, whereas 30.8% did not have children. Table 1 presents background information on the diagnosis of breast carcinoma in the experimental group.

**Table 1:** Descriptive statistics on diagnosis and currently treatment characteristics in the Experimental Group (*N* = 13) and Control Group (*N* = 12) of breast cancer survivors.

Variables	Experimental group	Control group
Past stage of diagnosis		
Metastatic stage	2	2
Non-metastatic stage	11	10
Current stage of disease		
Metastatic stage	–	–
Non-metastatic stage	13	12
Currently undergoing treatment		
Yes	7	5
No	6	7
Currently treatment typology (if answered yes to the previous question)		
Ormonal therapy	5	5
Radiotherapy	2	–
Chemiotherapy	–	–
Biological therapy	–	–
Last therapy		
Ormonal therapy	9	6
Radiotherapy	1	2
Chemiotherapy	1	2
Biological therapy	2	2
Presence of recurrences		
Yes	1	2
No	12	10
Menopausal stage at the time of diagnosis		
Pre-menopausal stage	1	4
Post-menopausal stage	4	1
Induced menopause for oncological treatment	1	1
No menopause	7	6

Due to the small sample size, frequencies are shown instead of percentages.

#### 2.1.2 Control sample

The control group comprised 12 breast cancer survivors, all recruited from the same hospital but who had not participated in any form of psychotherapy or psychological intervention. As the experimental group, they were invited to participate in this study via email or online post. However, women who belonged to the control group did not be involved in the psychological sessions; they only completed the questionnaires. The average age within this group was approximately 50.67 years, with a standard deviation of 9.40, indicating a slightly older cohort compared to the intervention group. Geographically, a plurality of the participants (41.7%) was from Northern Italy, followed by 33.33% from Central Italy, 16.7% from Southern Italy, and one individual residing in Alicante, Spain, providing a minor international dimension to the sample.

The educational attainment of the control group was notably varied: 8.3% had completed middle school education, 16.7% had a high school diploma, a significant 50% had earned a master’s degree, and 25% had acquired a doctoral degree, suggesting a highly educated cohort. Regarding marital status, half of the group (50%) were single, 41.7% were married, and 8.3% were separated. In terms of employment, the majority of the control group were active in the workforce, with only one participant identified as retired. Family configurations within the control group showed that 41.7% of participants had one or two children, whereas a larger percentage (58.3%) had no children, contrasting with the family structure observed in the intervention group. Considering its socio-demographic and illness characteristics, this control group offers a critical comparative baseline to assess the psychological intervention’s effects on breast cancer survivors. Table 1 presents background information on the diagnosis of breast carcinoma in the control group.

#### 2.1.3 Psychological intervention

Breast cancer survivors agreed to participate in the present project and in all its sessions. Online psychological group lasted 2 h weekly for a total of 4 sessions, in line with previous studies (Sebri et al., 2023a,b). Based on the results obtained, this study followed their contents, objectives, and timeline, aiming to provide new insights into a validated study process and methodology. Accordingly, it was conducted by a psychologist with an extensive expertise in oncological issues and BI particularly. The main contents proposed were focused on addressing BI to promote effective cognitions, emotions, and behaviors. Specifically, the present psychological intervention addressed risk factors of body dissatisfaction among breast cancer survivors, involving socio-cultural appearance pressure and the internalization of standards of beauty. Furthermore, emotional strategies to manage anxiety and depression were proposed, starting from a body compassion approach (Altman et al., 2017) based on a kind approach toward the own body. Particularly, the present intervention has been divided in four sessions that proposed the main following contents:

- Definition of body image and its related issues, aiming to make participants more confident about what body image is.
- The main emotions who are associated with body image issues, providing the opportunity to reflect about what are main emotional consequences of body dissatisfaction;
- The impact of body image on intimate and social relationship after oncological treatments; focusing on changes in significant relationship due to the cancer onset;
- Clinical strategies to address body image issues, proposing a new approach to one’s own body, namely body compassion.

To this aim, participants were invited to share their illness experiences and opinion in the group, exploiting body image issues and its related thoughts and emotions. Furthermore, psychologist proposed in-group activities and take-home tasks (e.g., taking note of emotions and thoughts in a daily diary). As shown in the literature (Bosch et al., 2020), the benefits of a diary are likely to reflect the amount of day-to-day emotions and their occurrences, focusing on participants’ feeling in real time. The present program was proposed online to increase participation all over Italy, being a cost-effective way (Esplen and Trachtenberg, 2020). At the same time, sharing emotions and discussing BI issues in a group of peers is beneficial for breast cancer survivors, thanks to a positive influence of newly social connections with women who dealing with similar issues (Durosini et al., 2021). As published by Durosini et al. (2021), addressing emotional issues in a group of breast cancer patients and survivors can be a positive experience supported by the promotion of newly social connections, which are sometimes damaged by illness. Moreover, perceiving cohesion can decrease anxiety, depression, and the lack of self-efficacy, common in women with a cancer diagnosis.

### 2.2 Instruments

#### 2.2.1 Body dissatisfaction in women who have had breast cancer

To measure participants’ body dissatisfaction, the Italian version (Cheli et al., 2016) of the Body Image Scale (BIS; Hopwood et al., 2001) was used. The BIS measures body dissatisfaction in women who have undergone medical treatment for a disabling illness. The scale consists of 10 items rated on a 4-point Likert scale (1 = not at all to 4 = very much) (e.g., “Have you felt less physically attractive because of the illness or treatment?”). High scores on the scale are an indication of high levels of body dissatisfaction. The Cronbach’s alpha of the scale in the present study was 0.88.

#### 2.2.2 Social physique anxiety

To measure participants’ social physique apprehension, the Italian version (Policardo et al., 2023) of the Social Physique Anxiety Scale 7 (Motl and Conroy, 2001) was used. The scale consists of 7 items (e.g., “In the presence of others I feel apprehensive about my physique/figure”) rated on a 5-point Likert scale (1 = not at all characteristics of me; 5 = extremely characteristic of me). High scores are related to high social physique anxiety, except for Item 5, which is formulated inversely (e.g., “I feel comfortable about how others evaluate my body”). The Cronbach’s alpha of the scale in the present study was 0.94.

#### 2.2.3 Functionality appreciation

To measure body-functionality appreciation, the Italian version (Cerea et al., 2021) of the Functionality Appreciation Scale (FAS; Alleva et al., 2017) was used. The scale consists of 7 items rated on a 5-point Likert scale (1 = strongly disagree to 5 = strongly agree) (e.g., “I feel that my body does a lot for me”). Higher scores indicate a high level of functionality appreciation. The Cronbach’s alpha of the scale in this study was 0.85.

#### 2.2.4 Body compassion

To measure body compassion, the Italian version (Policardo et al., 2021) of the Body Compassion Scale (BCS; Altman et al., 2017) was used. The scale measures body compassion (e.g., “When I think about my body’s imperfections, I tend to feel more separated and excluded from other people”) and consists of 23 items rated on a 5-point Likert scale (from 1 = almost never to 5 = almost always). Higher scores indicate greater body compassion. The Cronbach’s alpha of the scale in the present study was 0.78.

#### 2.2.5 Emotional regulation

Participants completed the Italian version (Balzarotti et al., 2010) of the Emotion Regulation Questionnaire (ERQ) (Gross and John, 2003). This instrument assesses emotional regulation strategies through two primary constructs: Expressive Suppression and Cognitive Reappraisal. Individuals rated their agreement with statements such as “I try to keep my emotions to myself” (for Expressive Suppression) and “I change what I’m thinking about to feel better” (for Cognitive Reappraisal) on a 7-point Likert scale (1 = Strongly Disagree, 7 = Strongly Agree). The internal reliability of the ERQ in the present study was assessed using Cronbach’s alpha (=0.68).

#### 2.2.6 Questions related to breast cancer

To gather information regarding the diagnosis and treatment received by both groups of women, specific inquiries were made. Participants were asked about the year of diagnosis and the cancer stage at the time of diagnosis. Additionally, details were sought about past and current treatments, occurrences of recurrence, and menopausal status at the time of the survey. The answers to these questions were used to provide descriptive data that helped us to ensure that the experiences and characteristics of the diseases were similar in both groups, which was crucial given our small sample size.

#### 2.2.7 Body mass index

Participants reported their height and weight; these self-reported data were used to calculate BMI (kg/m^2^).

#### 2.2.8 Sociodemographic details

Participants also provided their year of birth (from which their age was calculated), nationality, place of residence, education level, marital status, and occupation.

### 2.3 Data analysis

The data collected were analyzed using IBM© SPSS© Statistics version 20 (IBM© Corp., Armonk, NY, USA). Descriptive statistical methods were initially applied to examine the general characteristics of the sample and the variables of interest. The normality of the data was assessed using standard observational and statistical methods (skewness and kurtosis value). Given the slightly non-normal distribution of the scores and the small sample size, we opted for the Mann–Whitney *U* test to compare the distributions of scores between the groups across various secondary outcome measures (body dissatisfaction, social physique anxiety, functionality appreciation, body compassion, and emotional regulation). This non-parametric test was chosen as it does not assume normal distribution of the data, making it more suitable for our dataset. The analysis through the Mann–Whitney *U* test allowed for a more appropriate evaluation of the differences between the experimental group and the control group. A *p*-value of less than 0.05 was considered statistically significant. The effect sizes (*r*) were interpreted using the cut-off values proposed by Cohen (1988), where 0.1 indicates a small effect, 0.3 a medium effect, and 0.5 a large effect.

## 3 Results

Descriptive analyses of information on cancer diagnosis, treatment, recurrence, and related factors are presented in Table 1. The results show that the experimental and control groups of breast cancer survivors had similar characteristics regarding diagnosis and treatment, including past stage of diagnosis, current stage of disease, current treatment, presence of recurrence, and last therapy. This similarity in disease experience provides the basis for comparing the two groups on key study variables.

The descriptive analyses concerning the scores obtained on the variables under study, including positive and negative body image and emotional regulation, are presented in Table 2. The median score is reported as a descriptive point of reference, given that the sample sizes are relatively modest (13 and 12 participants), we propose consider medians as a more reliable measure of central tendency, thereby reducing the potential bias exerted by outliers.

**Table 2:** Descriptive statistics for each study’s variable and Mann–Whitney *U* test results for experimental (*N* = 13) and Control (*N* = 12) Groups of breast cancer survivors.

Variable	Group	Median (range)	*U* value	*p*-value	*Z* value
SPAS	Experimental	3.71(1.86–5.00)	34.00	0.016	−2.40
	Control	2.14 (1.29–3.86)			
BIS	Experimental	24 (10–30)	58.50	0.294	−1.06
	Control	14.50 (5–30)			
BCS	Experimental	2.82 (1.78–3.78)	86.50	0.649	0.46
	Control	2.74 (2.26–3.96)			
FAS	Experimental	3.57 (2.86–4.43)	143.50	0.000	3.57
	Control	4.36 (3.98–5)			
ERQ_Creapp	Experimental	16 (12–21)	28.00	0.005	−2.73
	Control	13.5 (4–18)			
ERQ_Esupp	Experimental	26 (19–38)	118.00	0.029	2.18
	Control	33.50 (25–42)			

To analyze the statistical differences between the groups, the Mann–Whitney *U* test was employed. Given the small sample sizes and some deviations from normality, this non-parametric test was appropriate. For example, cognitive reappraisal in the control group showed a significant deviation from normality with a skewness of –0.368 (SE = 0.637) and a kurtosis of –1.717 (SE = 1.232), and the Shapiro–Wilk test for this variable yielded *p* = 0.032, confirming the deviation from normal distribution.

**Figure 1:**
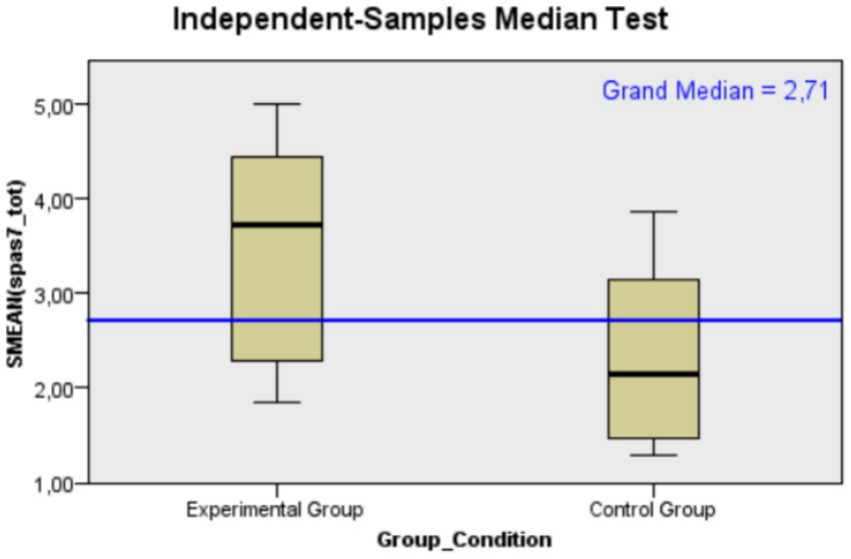
Mann–Whitney *U* test distribution of social physique anxiety’ scores for Control (*N* = 12) and Experimental (*N* = 13) groups, indicating a higher median score in the Experimental group.

Starting from *negative body image*, as can be seen in Table 2, the experimental group showed a median SPAS’s score of 3.71 compared to 2.14 in the control group, suggesting a greater concern about one’s physical appearance in social contexts in the experimental group (*U* = 34.00, *p* = 0.016, *r* = 0.480). This difference is statistically significant, with a medium effect size (for a graphical representation of the median differences between the two groups, see Figure 1). In terms of body dissatisfaction related to the oncological experience, the experimental group had a median BIS score of 24 compared to 14.50 in the control group, indicating a higher level of body image dissatisfaction in the experimental group (*U* = 58.50, *p* = 0.294, *r* = 0.212). However, this difference is not statistically significant.

**Figure 2:**
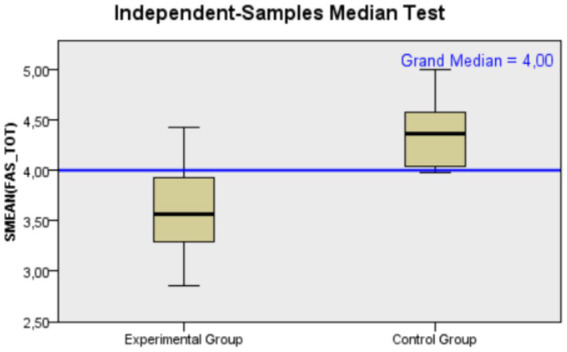
Mann–Whitney *U* test distribution of functionality appreciation’ scores for Control (*N* = 12) and Experimental (*N* = 13) groups, indicating a higher median score in the Control group.

Moving to *positive body image* results, median scores for body compassion were comparable between the two groups, with the experimental group at 2.82 and the control group at 2.74, indicating similar levels of self-compassion for the body (*U* = 86.50, *p* = 0.649, *r* = 0.092). This difference is not statistically significant. Regarding to functionality appreciation the control group showed a higher median score for this variable with a value of 4.36 compared to 3.57 for the experimental group, suggesting a higher appreciation for body functionality in the control group (*U* = 143.50, *p* < 0.001, *r* = 0.714). This difference is statistically significant, with a large effect size (for a graphical representation of the median differences between the two groups, see Figure 2).

**Figure 3:**
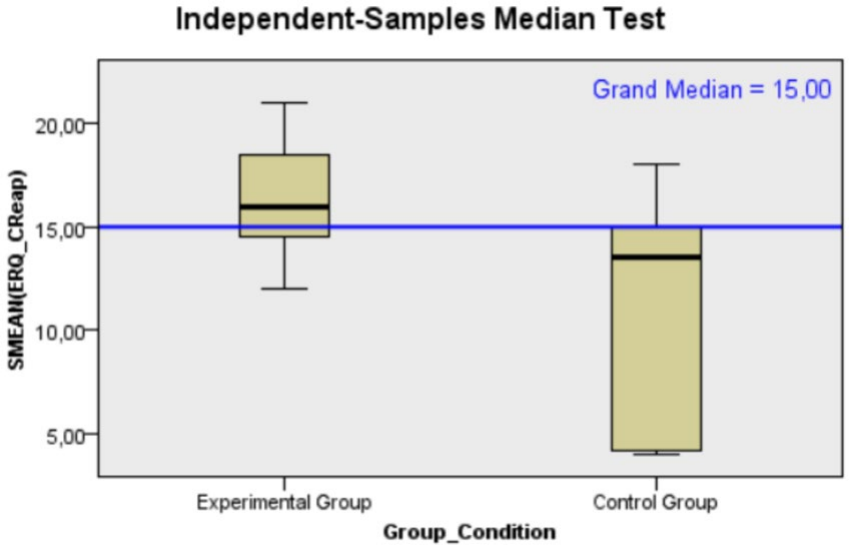
Mann–Whitney *U* test distribution of cognitive reappraisal’ scores for Control (*N* = 12) and Experimental (*N* = 13) groups, demonstrating a higher median score for the Experimental group.

**Figure 4:**
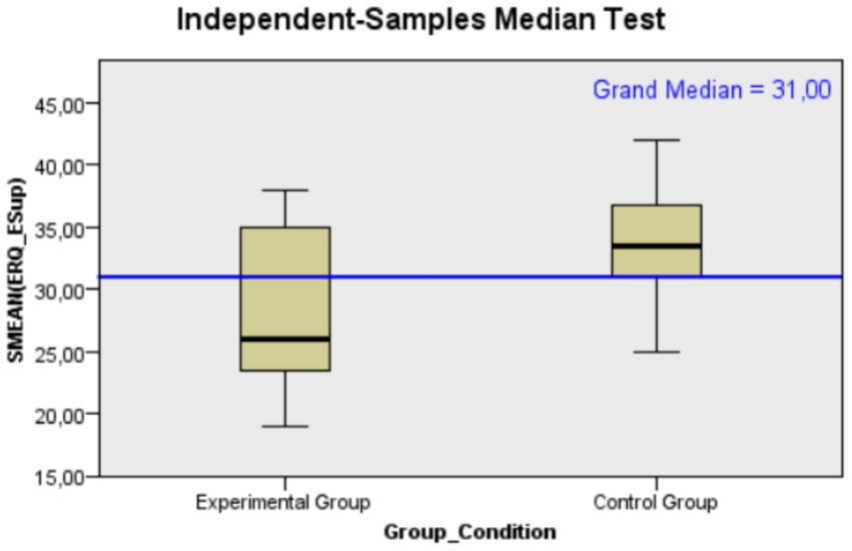
Mann–Whitney *U* test distribution of expressive suppression’ scores for Control (*N* = 12) and Experimental (*N* = 13) groups, showing a higher median score for the Control group.

Moving on to results related to *emotional regulation* regarding to cognitive reappraisal, the median score for the experimental group was 16, whereas it was 13.5 for the control group. This indicates a greater engagement in cognitive restructuring strategies in the experimental group (*U* = 28.00, *p* = 0.005, *r* = 0.546). This difference is statistically significant, with a large effect size (for a graphical representation of the median differences between the two groups, see Figure 3). About expressive suppression, the experimental group exhibited lower scores in this dimension with a median of 26 compared to 33.50 in the control group. This suggests a tendency toward reduced expressive suppression in the experimental group (*U* = 118.00, *p* = 0.029, *r* = 0.436). The observed difference is statistically significant, with an effect size that can be considered medium (for a graphical representation of the median differences between the two groups, see Figure 4).

## 4 Discussion

In the present study, we examined differences among groups of women who have undergone the highly complex experience of breast cancer. Specifically, our focus was on investigating whether differences exist between women who have experienced breast cancer and did not participate in any psychological intervention versus those who engaged in a brief online psychological intervention centered on compassion toward their own bodies. We aimed to explore differences between groups in terms of both positive and negative evaluations of their body image (Hp1) and emotional regulation abilities (Hp2).

Hypothesis 1 suggested that the experimental group would experience higher positive body evaluation aspects and reductions in negative aspects. The results offer a mixed support for this hypothesis. This is in line with the relevant contents focused on body image within the psychological interventions. While the experimental group exhibited unpredicted higher levels of social physique anxiety and the control group demonstrated higher levels of functionality appreciation.

The higher levels in social physique anxiety observed in the experimental group (respect to control group) could be explained by the intervention’s focus on BI post-cancer potentially heightening participants’ awareness and sensitivity to their physical selves. Discussing BI and its challenges may inadvertently magnify concerns about appearance, as the literature suggests that focused attention on specific self-aspects can temporarily heighten self-criticism and sensitivity to external evaluations, especially in contexts where physical appearance is perceived as altered or compromised (Cash et al., 2004; Fingeret et al., 2014). Moreover, discussing cancer-related body image issues with others may enhance self-referential effects in metacognitive sensitivity, which is closely linked to body image concerns (Dent and Martin, 2023). This phenomenon underscores the delicate balance required in interventions addressing BI, highlighting the need for approaches that carefully navigate the enhancement of positive BI without unintentionally reinforcing negative perceptions. Moreover, being expose to the others’ judgments could have increased anxiety and distress for the fear of negative opinions (Oren-Yagoda et al., 2024). In a study by Asher and Aderka (2021), feelings of shame could hide individuals’ true self, increasing social anxiety and poor relationship. In this way, the present study highlighted the relevance of self-disclosure in psychological interventions, where women with similar experiences of illness can share their emotions and thoughts. However, it is paramount to guarantee the presence of ax expert psycho-oncologist allowing for benefits thanks to the promotion of social connections (Durosini et al., 2021; González Alsina, 2023).

Unexpected finding that the control group exhibited higher levels of functionality appreciation suggests that interventions might, paradoxically, lead to increased concerns about body functionality among participants who engage with BI content. This could be attributed to the intervention making participants more aware of their bodies, thereby inadvertently focusing their attention on functional limitations or changes post-cancer treatment. The health psychology literature emphasizes that increased health-related self-focus can lead to a heightened perception of symptoms and limitations (Pennebaker and Skelton, 1978), which may probably explain the observed pattern in functionality appreciation. Accordingly, another possible interpretation is that becoming more aware of one’s own body can lead to fears, anxieties, and worries. Therefore, it is crucial to offer effective psychological support to manage these new sensations and emotion, sustaining the abilities to integrate their body and emotions (Qu et al., 2023).

The outcomes of this study, particularly the increased social physique anxiety and the unexpected findings in functionality appreciation, align with metanalysis conducted by Rodrigues et al. (2023), which underscores the complexities of improving BI post-cancer. The necessity for women to navigate the transition to a new BI post-cancer, often requiring a reconciliation with the loss of their pre-cancer image, might contribute to heightened concerns around physical appearance and functionality observed in interventions.

The second hypothesis of our study posited that the experimental group would exhibit higher emotional regulation, characterized by lower expressive suppression and higher cognitive reappraisal, compared to the control group. The results obtained from the Mann–Whitney *U* test substantiate this hypothesis, revealing statistically significant differences in the realms of cognitive reappraisal and expressive suppression between groups. This finding, supported by a substantial effect size, aligns with previous literature indicating the potential of targeted interventions to enhance cognitive reappraisal abilities among individuals facing psychological distress after breast cancer treatment (Gross and John, 2003). Additionally, it is needed to consider that high level of cognitive reappraisal in the experimental group could be the result of the psychological intervention, in which participants discussed about new meanings about breast cancer. In this regard, can bring up differ perspectives, allows for new explanations and interpretations about one’s own experience.

Conversely, the control group exhibited a higher tendency toward expressive suppression pointing to a relative reduction in the use of such behaviors among the experimental group participants. As affirmed by Gross and John (2003), expressive suppression may be associated with a maladaptive strategy that interfere the expression negative emotions, such as anxiety and distress. In line with this perspective, individuals lacking the opportunity to express their emotions in a supportive context (e.g., group intervention conducted by an expert) might tend to suppress dysfunctional feelings fearing they may be unable to effectively manage them. Moreover, existing literature substantiates the effectiveness of psychological interventions, particularly those incorporating behavioral skill training, in mitigating cognitive distortions among cancer survivors (Asher et al., 2019; Cherrier et al., 2022). In line with this, the current brief online psychological program encouraged participants to focus their attention on their bodies and emotions and promoted open discussions within the group. This approach is consistent with the promotion of cognitive elaboration and restructuring of thoughts and cognitions, particularly in relation to emotions and the ability to be aware of and manage them (Jelvehzadeh et al., 2022). The observed improvements in the cognitive reappraisal component suggest that the intervention successfully facilitated cognitive strategies for emotional regulation in breast cancer survivors (Figure 4).

## 5 Conclusion

The findings from this investigation highlight the nuanced outcomes associated with a cost-effective brief psychological intervention focused on BI concerns among breast cancer survivors. Results allow for preliminary interpretations about the potential link between a brief psychological intervention centered on BI and observable changes in aspects of functional emotional regulation among breast cancer survivors, cognitive reappraisal in particular. This is in line with literature that evidence the functional association of a body compassion approach with cognitive reappraisal strategies to promote well-being, reducing shame-proneness and irrational beliefs for instance (Cȃndea and Szentágotai-Tătar, 2018). While the absence of pre-intervention measurements in this study is acknowledged, the deliberate focus on post-intervention assessments among breast cancer survivors who underwent psychological therapy distinguishes its design. Moreover, the comparison of outcomes between participants who received psychological intervention and those without any psychological intervention provides a targeted examination of the intervention’s immediate impact. This approach yields preliminary insights into distinctive changes attributable to psychological therapy within the survivor population, which need to be better explored. Finally, by centering on post-intervention measurements, the study preliminary explores the impact of psychological interventions in fostering positive outcomes. This focus not only offers timely and insights for clinical applications, but also provides suggestions that have to be considered to assess the intervention’s impact in a real-world context. A noteworthy finding in this study is the difference observed in emotional regulation, where women who participated in the intervention demonstrated significantly higher scores compared to the control group. The observed difference in the cognitive reappraisal and expressive suppression highlights the potential therapeutic impact of the intervention on emotional regulation strategies. Accordingly, the current literature emphasizes the relevant role of psychological intervention that as promising to improve and maintain emotional regulation in cancer survivors (Smith et al., 2023). Considering cancer onset and its diagnosis as a traumatic event (Wilson, 2020), women need for time to promote their identity reconstruction within a well-structured psychological intervention.

However, cautious interpretations are needed, given the study’s methodological constraints. The exploration into the intervention’s outcomes suggests a promising direction for enhancing emotional regulation, though it stops short of establishing causality. Future research, with more rigorous and structured protocols, is needed to deepen our understanding of how such interventions might support the emotional wellbeing of breast cancer survivors. Additionally, to enhance the generalizability of these observations, future studies could benefit from expanding participant samples. While this investigation made efforts to maintain comparability between the experimental and control groups, the relatively small overall sample size introduces limitations. Increasing the sample size would serve to bolster the statistical validity of the findings and offer a richer insight into the intervention’s effects across varied demographic and clinical characteristics. Accordingly, comparing 13 (experimental group) and 12 women (control group) presents a limitation; future research should enlarge the sample size to allow for more robust comparisons. Additionally, efforts should be made to ensure the two groups are more homogeneous, particularly regarding socio-demographic characteristics not included in this study. For instance, motherhood is a crucial variable to consider due to its relationship with cancer and oncological treatments. Achieving balanced and comparable groups can be facilitated through a randomized and counterbalanced recruitment process, which will also enhance the study’s replicability. An additional limitation arises from the absence of pre-intervention measurements for the assessed variables. The lack of baseline data poses challenges in comprehensively understanding participants’ initial states, thereby hindering the ability to attribute observed changes solely to the intervention. This limitation compromises the study’s capacity to establish causal relationships and impedes a nuanced interpretation of the therapeutic effects over time. Future research endeavors should prioritize the inclusion of pre-intervention assessments to address this limitation and contribute to a more robust understanding of the intervention’s impact on participants’ emotional well-being. Accordingly, predictive analyses and other participatory methods to explore patients’ needs and experiences could to be conducted in future research. Moreover, future studies should involve a follow-up phase to assess the long-term outcomes of the results with a focus on longitudinal analysis. Furthermore, future research could employ a quanti-qualitative study design to better address patients’ needs and more precisely explore the goals of the intervention.

In conclusion, the present study provides promising findings to address insight into the specific psychological mechanisms affected by the therapeutic approach. They can contribute to the growing body of knowledge on the role of psychological interventions in addressing emotional regulation challenges in breast cancer survivors. Exploiting clinical implications, findings suggest that BI and its related issues could be strictly considered as main contents to address with breast cancer survivors. Therefore, future interventions for breast cancer survivors should incorporate BI and its implications, with a focus on the emotions and behaviors closely tied to BI changes. Additionally, psycho-oncologists should be trained to address BI concerns and recognize their importance for breast cancer survivors.

## Data Availability

The raw data supporting the conclusions of this article will be made available by the authors, without undue reservation.
